# Hybrid Functional Brain Network With First-Order and Second-Order Information for Computer-Aided Diagnosis of Schizophrenia

**DOI:** 10.3389/fnins.2019.00603

**Published:** 2019-06-14

**Authors:** Qi Zhu, Huijie Li, Jiashuang Huang, Xijia Xu, Donghai Guan, Daoqiang Zhang

**Affiliations:** ^1^College of Computer Science and Technology, Nanjing University of Aeronautics and Astronautics, Nanjing, China; ^2^Collaborative Innovation Center of Novel Software Technology and Industrialization, Nanjing, China; ^3^Department of Psychiatry, Affiliated Nanjing Brain Hospital, Nanjing Medical University, Nanjing, China; ^4^Department of Psychiatry, Medical School, Nanjing Brain Hospital, Nanjing University, Nanjing, China

**Keywords:** brain network, second-order information, rs-fMRI, computer-aided diagnosis, schizophrenia classification

## Abstract

Brain functional connectivity network (BFCN) analysis has been widely used in the diagnosis of mental disorders, such as schizophrenia. In BFCN methods, brain network construction is one of the core tasks due to its great influence on the diagnosis result. Most of the existing BFCN construction methods only consider the first-order relationship existing in each pair of brain regions and ignore the useful high-order information, including multi-region correlation in the whole brain. Some early schizophrenia patients have subtle changes in brain function networks, which cannot be detected in conventional BFCN construction methods. It is well-known that the high-order method is usually more sensitive to the subtle changes in signal than the low-order method. To exploit high-order information among brain regions, we define the triplet correlation among three brain regions, and derive the second-order brain network based on the connectivity difference and ordinal information in each triplet. For making full use of the complementary information in different brain networks, we proposed a hybrid approach to fuse the first- and second-order brain networks. The proposed method is applied to identify the biomarkers of schizophrenia. The experimental results on six schizophrenia datasets (totally including 439 patients and 426 controls) show that the proposed method outperforms the existing brain network methods in the diagnosis of schizophrenia.

## Introduction

Resting-state functional magnetic resonance imaging (rs-fMRI) studies indicate that there exists a disorder-related alteration in BFCN (Bluhm et al., 2007; Jafri et al., 2008; Fornito and Bullmore, 2010; Shafiei et al., 2018; Wang et al., 2018). As a severely debilitating mental illness, schizophrenia is usually thought of as connectivity disorders between brain regions (Liang et al., 2006; Salvador et al., 2010). In recent years, many BFCN analysis methods (Zhou et al., 2006; Liang et al., 2007; Honey et al., 2009; Tsuang et al., 2009) have been proposed to explore the biomarkers for schizophrenia. Most of the existing methods first construct the brain functional network by measuring the correlation of brain regions or voxels, and then perform feature extraction on the produced large-scale brain networks for selecting the significant features. These connectivities or sub-networks having significant alterations in some indicators, e.g., topological metrics (Fei et al., 2014) and the alteration degree (Guo et al., 2014; Zhu et al., 2018), are chosen as the biomarker for the disease.

The construction of the brain network plays as an important role in the diagnosis system. The previous BFCN construction methods mainly focus on revealing the low-order information among brain regions or voxels. In other words, the functional connectivities in these methods are usually denoted as the association between each pair of brain regions or voxels; e.g., the Pearson correlation (PC) based method uses the pair-wise linear correlation of brain regions as the connectivity strength (Richiardi et al., 2013). In addition, the Kendall correlation (KC) (Dong et al., 2014) and the Spearman correlation (SC) (Zhang et al., 2015) are also introduced into the brain network construction process. Considering the correlation between the two brain regions may be affected by other regions, Guo et al. (2014) proposed to eliminate this kind influence from other connectivities through the partial correlation (ParC) test, and applied it to the classification of schizophrenia patients and healthy controls. Li et al. (2019) and Qiao et al. (2016) constructed the low rank and self-weighted brain network by introducing prior knowledge to the model. Zhou et al. (2018) embedded the second-order information among brain regions into brain network and applied it into identifying mild cognitive impairment.

The low-order method has the following natural defects in characterizing the relationship among brain regions. On one hand, there exists the subtle alteration of brain network structure between controls and patients, especially the mild or early patients, which is hard to reveal through the first-order method because, compared to the high-order method, the low-order method is not sensitive to small signal change. On the other hand, the first-order or pair-wise model is not capable of describing the complex multivariable correlation and ignores the ordinal pattern among connectivities (Wu et al., 2018). The topological structure of the brain network is intricate (Bullmore and Sporns, 2009); e.g., one brain region usually interacts with multiple brain regions physiologically, whereas the low-order methods often ignore the relationship among multi-regions. Therefore, it is necessary to investigate effective high-order brain network construction methods to reveal the correlation of multiple brain regions and ordinal patterns from rs-fMRI data.

In this paper, we derived the second-order brain networks by introducing the triplet of brain regions. We calculated the connectivity difference to describe the ordinal information in each triplet. The derived second-order relationship in BFCN construction methods is more capable of capturing the changes in the brain network, making it easier to identify mild or early schizophrenia patients. Considering that the brain network has the property of high regional agglomeration (Alexander-Bloch et al., 2013), we extract the second-order relationship among one brain region and its *k* nearest neighbors to improve the robustness of the proposed method. Then, we fuse our second-order BFCN with the most widely used first-order BFCN, i.e., PC based brain network, to construct the hybrid functional brain network. The mixed model not only reveals the subtle differences in brain networks between patients and healthy controls, but also makes full use of complementary brain networks defined in different orders. Our proposed method has the following advantages:

1. We embed high-order information of connectivity groups in the brain network, which may have a beneficial effect on the analysis and diagnosis of mild or early mental illness due to the revelation of a more subtle alteration.
2. Considering the regional aggregation of brain networks, we utilize the local strategy to improve the robustness of the proposed second-order brain network.
3. A hybrid strategy fusing the proposed second-order brain network and the conventional first-order brain network is proposed, which can make full use of the complementary information in these two types of networks.
4. The experiment is performed on six rs-fMRI datasets with schizophrenia patients and controls, including a total of 865 subjects. To the best of our knowledge, it is with the largest population reported in the literature of rs-fMRI analysis for identifying schizophrenia. The results show that the proposed hybrid network is superior to the state-of-the-art methods.

The remainder of the paper is organized as follows: first, we introduce our materials and methods, i.e., the six schizophrenia datasets and the proposed brain network construction method. Then, the experimental results and discussion on six schizophrenia datasets are presented. Finally, we summarize our work and provide the conclusion.

## Materials and Methods

### Subjects

Six schizophrenia rs-fMRI datasets we used are Nanjing Brain Hospital (NBH) dataset, The Center for Biomedical Research Excellence (COBRE) dataset, Huaxi dataset, Nottingham dataset, Taiwan dataset, and Xiangya dataset. The subjects have the following requirements: (1) no other Diagnostic and Statistical Manual of Mental Disorders (DSM-IV) disease exists; (2) no history of drug abuse; (3) no clinically significant head trauma. Table 1 summarizes the demographic and clinical characteristics of participants of these datasets (Cheng et al., 2015). The Positive and Negative Symptom Scale (PANSS) (Kay et al., 1987) is used by experts to score participants to obtain the severity of schizophrenia.

**Table 1:** Demographic and clinical characteristics of participants in six schizophrenia datasets.

	**Group**	**Age**	***P*-value of age**	**Gender (M/F)**	***P*-value of gender**	**PANSS-positive scale**	**PANSS-negative scale**	**PANSS-general scale**	**PANSS-total**
NBH	Healthy	35.29 ± 7.94	0.0813	10/14	0.3600	–	–	–	–
	Patient	30.78 ± 9.01		6/15		–	–	–	97.83 ± 11.09
COBRE	Healthy	34.82 ± 11.28	0.3987	46/21	0.1927	–	–	–	–
	Patient	36.75 ± 13.68		42/11		14.84 ± 4.53	14.42 ± 4.97	29.88 ± 8.27	–
Huaxi	Healthy	27.80 ± 12.50	1.0000	95/85	0.6748	–	–	–	–
	Patient	27.80 ± 12.50		90/88		24.48 ± 6.05	19.68 ± 7.67	46.70 ± 8.87	–
Nottingh-am	Healthy	33.38 ± 8.98	0.9855	26/10	0.2277	–	–	–	–
	Patient	33.34 ± 9.05		27/5		3.84 ± 3.18	3.13 ± 3.63	5.94 ± 3.89	–
Taiwan	Healthy	29.87 ± 8.62	0.2847	25/37	0.2329	–	–	–	–
	Patient	31.59 ± 9.60		35/34		11.92 ± 4.71	13.61 ± 6.33	27.28 ± 9.64	52.81 ± 16.68
Xiangya	Healthy	27.17 ± 6.64	0.1025	35/25	0.9333	–	–	–	–
	Patient	23.37 ± 7.83		49/34		19.84 ± 6.31	21.56 ± 7.66	39.24 ± 11.75	–

### Image Acquisitions, Data Preprocessing and Anatomical Parcellation

For NBH, COBRE, Taiwan, and Xiangya datasets (Guo et al., 2014), the rs-fMRI images of each participant were acquired by using a 3-Tesla Siemens Tim-Trio scanner with an eight or 12 channel head coil. For Huaxi dataset, the rs-fMRI images were acquired by using a 3-T General Electric MRI scanner. For the Nottingham dataset, the images were acquired using a 3-T Philips Achieva MRI scanner.

During the scanning of images in the NBH, COBRE, and Nottingham datasets, all participants must keep their eyes open and stare intently at the fixed cross in the middle of the screen for 5 min. During scanning of images in the Huaxi, Taiwan, and Xiangya datasets, the participants were instructed to keep their eyes closed but not fall asleep.

For all datasets to be preprocessed, we discarded the first 10 volumes to make sure scanner stabilization and the subjects' adaptability to the environment. Rs-fMRI data preprocessing was then performed by statistical parametric mapping (SPM8) software (http://www.fil.ion.ucl.ac.uk/spm) and a Data Processing Assistant for RS-fMRI (DPARSF). The remaining functional scans were first corrected to the difference in acquisition time between on-chip scans, and then readjusted to the intermediate volume to correct for head movement between scans. The functional scan was then spatially normalized to the Montreal Neurological Institute template and resampled to 3 × 3 × 3 mm^3^ voxels. After linear detrending, data was filtered using typical temporal bandpass (0.01–0.08 Hz) to reduce low-frequency drift and high-frequency physiological noise. Next, four covariates of no interest (i.e., six rigid-body motion parameters, the global mean signal, white matter, cerebrospinal fluid) were regressed from the data. No subject was excluded under a head motion criterion of 3 mm and 3°.

After data preprocessing, according to Lynall et al. (2010), the rs-fMRI images were divided into 90 brain regions (excluding the cerebellum) or region-of-interests (ROIs) using the Anatomical Automatic Labeling (AAL) template (Tzourio-Mazoyer et al., 2002). Finally, for each ROI, the average rs-fMRI time series over all voxels was taken as the time series for that ROI. After data preprocessing and anatomical parcellation, for each of the brain regions of each subject, its feature information is represented in the form of the time series. The processed information of all datasets is shown in Table 2.

**Table 2:** The processed information of all six schizophrenia datasets.

**Dataset**	**# of patient samples**	**# of normal samples**	# of features (brain regions * time series)
NBH	24	21	90*230
COBRE	53	67	90*145
Huaxi	178	180	90*180
Nottingham	32	36	90*235
Taiwan	69	62	90*170
Xiangya	83	60	90*170

### Triplet-Based Second-Order Functional Brain Network

The traditional functional brain network methods often only consider the pairwise relationship of different brain regions, namely the first-order relationship. However, the alteration of connectivity networks between patients and healthy controls may be subtle, and the first-order brain network is not capable of finding the subtle difference. In addition, the topology of the brain is extremely complex and one brain region usually interacts with multiple brain regions. For revealing the high-order information among brain regions, we construct the second-order brain network by introducing triplet correlation among three brain regions.

Suppose that $X=\left\{x_{1},\;x_{2},\;\ldots ,\;x_{n}\right\}\in R^{n\times m}$ denotes the rs-fMRI data from a subject, where *n* is the number of brain regions and *m* is the number of time series. *x*_*i*_ is the time series for the *i*-th brain region where *i* ∈ {1, 2, … , *n*}. Our triplet-based second-order functional brain network is constructed as follows:

Let triplet (*x*_*i*_, *x*_*u*_, *x*_*v*_) consists of *x*_*i*_ and its neighbors *x*_*u*_ and *x*_*v*_. $S_{uv}^{i}$ defines the distance between *x*_*i*_ and *x*_*v*_ relative to *x*_*u*_:

(1) $$\begin{array}{r} S_{uv}^{i}=dist\left(x_{i},x_{v}\right)-dist\left(x_{i},x_{u}\right) \end{array}$$

where *dist*(·, ·) denotes the squared Euclidean distance. It should be pointed out that *dist*(·, ·) has nothing to do with the anatomical distance, because *x*_*i*_ denotes the rs-fMRI time series data of the *i*-th brain region. Obviously, *S*^*i*^ is an antisymmetric matrix. Since one brain region usually interacts with its neighbors rather than distant brain regions, we only consider the *k* nearest neighbors of *x*_*i*_. Let *N*_*i*_ be a set of sequence numbers indicating the *k* nearest neighbors of *x*_*i*_, and then, relative to all *k* nearest neighbors of *x*_*i*_, the distance between *x*_*i*_ and *x*_*v*_ can be expressed as:

(2) $$\begin{array}{r} dist^{\prime }\left(x_{i},x_{v}\right)=\underset{u\in N_{i}}{\sum }S_{uv}^{i} \end{array}$$

Based on the above distance, the triplet correlation coefficient (Guo et al., 2017) between *x*_*i*_ and *x*_*v*_ can be expressed as:

(3) $$\begin{array}{r} C_{iv}=norm\left(dist^{\prime }\left(x_{i},x_{v}\right)\right) \end{array}$$

where *norm*(·) denotes normalizing the data to the interval [0, 1].

Thus, the second-order correlation coefficient matrix can be denoted as:

(4) $$C_{ij}=\{\begin{array}{c} \begin{array}{c} \begin{array}{c} norm\left(-dist^{\prime }\left(x_{i},x_{j}\right)\right),\left(j\in N_{i}\right)\\ 0,\left(j\notin N_{i}\right) \end{array} \end{array} \end{array}$$

Then, we symmetrically handle *C* as follows:

(5) $$\begin{array}{r} C^{\prime }=\frac{C\;+\;C^{T}}{2} \end{array}$$

**Figure 1:**
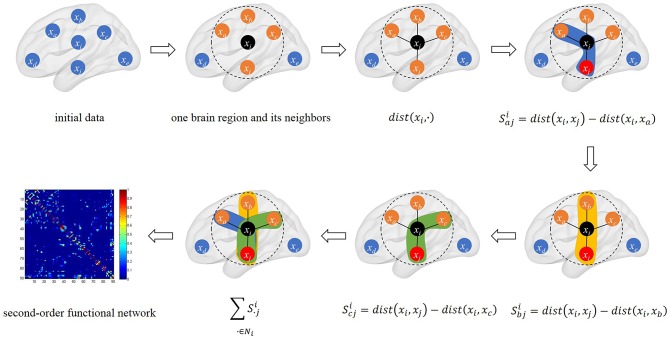
The schematic diagram of triplet-based local brain network. The black point *x*_*i*_ is the center point we selected. We calculated the distance between *x*_*i*_ and its neighbors in connectivity strength. There are three triples based on *x*_*i*_ and its neighbors in calculation. The last sub-graph is our triplet-based second-order functional network in the whole brain.

The schematic diagram of triplet-based local brain network is shown in Figure 1.

After all correlation coefficients are calculated, a second-order functional brain network is constructed. It preserves second-order information among brain regions, which makes it more sensitive to the changes in the brain network and can capture the ordinal information among connectivities. It can also be seen from the above formula that our second-order functional brain network will eventually be converted into a two-dimensional form, which also helps to reduce the space complexity of high-order method.

### Hybrid Functional Brain Network With First-Order and Second-Order Information

Studies have shown that brain network methods based on correlation coefficients can characterize the interactions between brain regions or neurons (Smith et al., 2011). The conventional first-order method and the proposed second-order method can construct connectivities from different levels. There is some complementary information in these two kinds of brain networks for the diagnosis task. In addition, the low-order method is more robust to noise transmission. Inspired by the above issue, we fuse the proposed brain network with a first-order brain network to construct a hybrid brain network. The PC method is a classical first-order method for constructing brain networks based on correlation coefficients. PC can be calculated using the following formula:

(6) $$\begin{array}{r} C_{ij}^{1}=\frac{Cov\left(x_{i},\;x_{j}\right)}{\sqrt{Var\left(x_{i}\right)Var\left(x_{j}\right)}} \end{array}$$

where *Var*(·) means a function of the variance calculated and *Cov*(·, ·) is a function that calculates the covariance.

**Figure 2:**
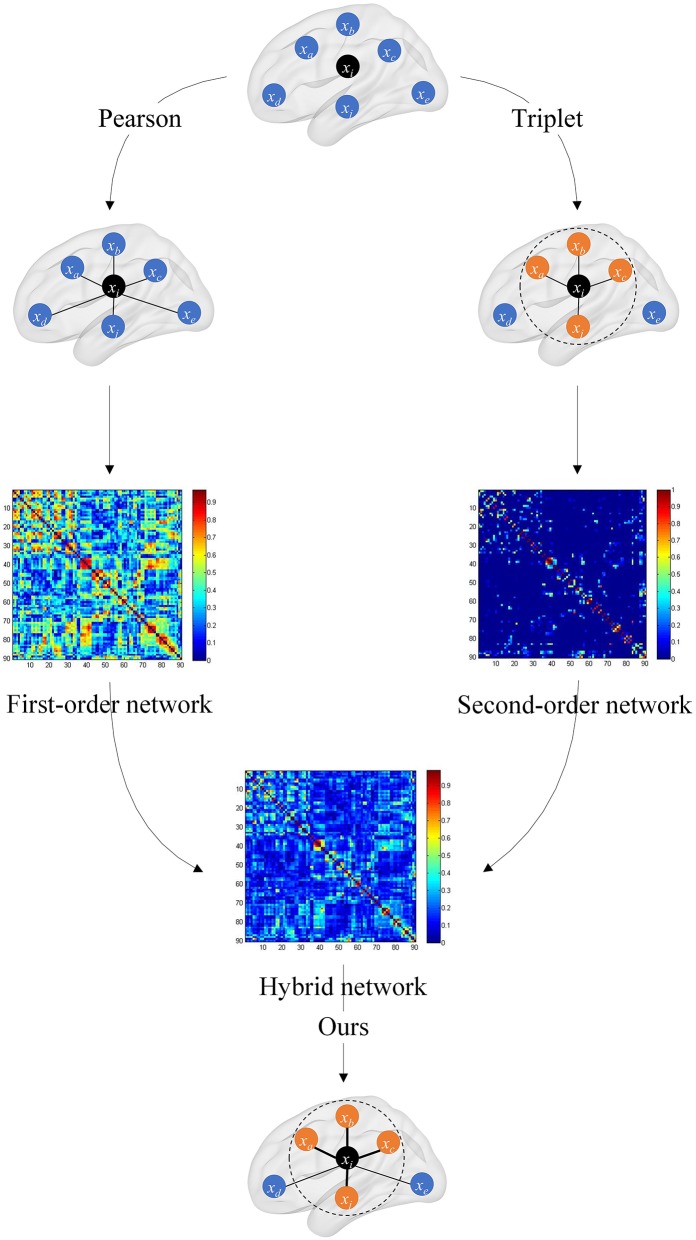
Overall framework of the hybrid brain network. The first-order brain network based on the Pearson correlation coefficient and the second-order brain network based on the triplet are, respectively, constructed, and then the two are fused with a certain weight to obtain the hybrid functional brain network with first- and second-order information.

For convenience, we denote the proposed second-order functional brain network *C*′*C*′ mentioned in the previous section as *C*^2^. In this case, hybrid functional brain network with first-order and second-order information can be expressed as:

(7) $$\begin{array}{r} C=\mu C^{1}+\left(1-\mu \right)C^{2} \end{array}$$

where μ is the weighted coefficient for mixing *C*^1^ and *C*^2^, and its ranges from 0 to 1. The overall framework of the hybrid brain network is shown in Figure 2.

In the hybrid brain network, *C*^1^ preserves the original connectivity strength information between the two brain regions, and *C*^2^ captures the connectivity strength information of one brain region and its neighbors. The hybrid brain network *C* synthesizes *C*^1^ and *C*^2^, and fuses the complementary information in these two kinds of networks.

By introducing the triplet correlation, the ordinal relationship among brain regions is preserved. Through second-order brain network, i.e., the triplet-based brain network, not only high-order information is introduced to extract neighbor ranking information in each brain region, but also connectivity noise is transmitted. From the point of view of signal analysis, the first-order brain network is robust to noise, and the second-order brain network is sensitive to the subtle differences between the brain network of patients and healthy controls. Thus, the proposed hybrid model can balance robustness and classification performance.

### Feature Selection Strategy

There are mainly three stages of mental illness classification based on BFCN: brain network construction, brain network feature selection, and classification. Besides the construction of functional brain networks, feature selection is also a key step in the brain network analysis process. Since this work focuses on brain network construction, the discussion of feature selection algorithm is beyond the research content of this paper. By comparing the classification performance of multiple feature selection algorithms, we use the non-negative elastic-net (Zhu et al., 2018) in our previous work to choose the significant connectivities.

The functional brain network data are high dimensional, and many subjects are linearly inseparable. To achieve good classification performance, we utilize kernel discriminant analysis (KDA) algorithm to project each sample to the feature space, in which the classification performance of subjects can be improved. Finally, the nearest neighbor classifier is exploited for making the diagnosis decision.

In addition, the topological properties of the brain network can also be used as important features. Similarly, since this work focuses on the construction of the brain network, we use the methods proposed by Narula et al. (2017) and the public code they provided to calculate the topological properties of the brain network.

## Experiments and Results

### Classification Performance of Different fMRI Functional Brain Networks

In this experiment, the brain networks were constructed using the proposed method and a series of comparison methods mentioned below. Since this experiment is designed for comparing the performance of different functional brain networks, the same feature selection and classification algorithm in our method are all performed on these networks to ensure the comparability of the functional brain networks.

#### Comparison Methods

We have chosen the following functional brain network construction methods as comparison methods: PC-based method (Richiardi et al., 2013), KC-based method (Dong et al., 2014), SC-based method (Zhang et al., 2015), ParC-based method (Tao et al., 2011), Gaussian Graphical Models (GGM) (Belilovsky et al., 2015), low order partial correlation (LOPC) (Zuo et al., 2014), and multi-threshold brain network based classification (MTNC) (Fei et al., 2014).

#### Experimental Setup

We adopt the 10-fold cross-validation strategy in this experiment. Specifically, each dataset is equally divided into 10 subsets. One subset is selected as the test set successively, and all remaining subsets are used for training. To avoid possible deviations during sample segmentation, we repeat this process 20 times. Accuracy (ACC), sensitivity (SEN), specificity (SPE), positive predictive value (PPV), negative predictive value (NPV), F1-score (F1), class balanced accuracy (BAC), the area under the receiver operating characteristic curve (AUC) and their respective standard deviations (STD) are employed to measure the performance in classification. They can be calculated as follows:

(8) $$\begin{array}{r} ACC=\frac{TP+TN}{TP+FN+TN+FP} \end{array}$$

(9) $$\begin{array}{r} SEN=\frac{TP}{TP+FN} \end{array}$$

(10) $$\begin{array}{r} SPE=\frac{TN}{TN+FP} \end{array}$$

(11) $$\begin{array}{r} PPV=\frac{TP}{TP+FP} \end{array}$$

(12) $$\begin{array}{r} NPV=\frac{TN}{FN+TN} \end{array}$$

(13) $$\begin{array}{r} F1=2\times \frac{SEN\times PPV}{SEN+PPV} \end{array}$$

(14) $$\begin{array}{r} BAC=\frac{SEN+SPE}{2} \end{array}$$

(15) $$\begin{array}{r} AUC=\frac{\sum_{i\in PatientClass}rank_{i}-\frac{N_{pat}\left(1+N_{pat}\right)}{2}}{N_{pat}\times N_{nor}} \end{array}$$

where TP, TN, FP, and FN are the number of correctly predicted patients, correctly predicted healthy controls, healthy controls predicted as patients, and patients predicted as healthy controls, respectively. *N*_*pat*_ and *N*_*nor*_ are the number of patient samples and the number of healthy control samples, respectively. *rank*_*i*_ represents the serial number of the *i*-th sample. In addition, we calculated the *p*-values (DeLong et al., 1988) of the AUC to measure statistically significant differences between proposed method and comparison methods. If the *p*-value is <0.05, it indicates that the increase in performance of our method compared to the comparative method is statistically significant (Wang et al., 2019).

For fair comparison, for our method, we adopt grid search to select the parameter of the number of neighbors from {5, 10, 15, … , 45} to specify the size of the neighborhood. The proportion weighted coefficients of the first-order functional brain network and the second-order functional brain network are selected from {0, 0.1, 0.2, … , 1} using greedy strategy. For ParC, GGM, and LOPC, ‘thres’ [a parameter for picking out statistically significant pairwise association for 0-th-order partial correlation and first-order partial correlation after correcting multiple testing problem using false discovery rate (FDR)] is selected from {0.0001, 0.001, 0.01, 0.05, 0.1, 0.2} using greedy strategy. In addition, we set the threshold from {0, 0.05, 0.1, … , 0.4}{0, 0.05, 0.1, … , 0.4} for all functional brain network methods.

#### Results and Analysis

**Figure 3:**
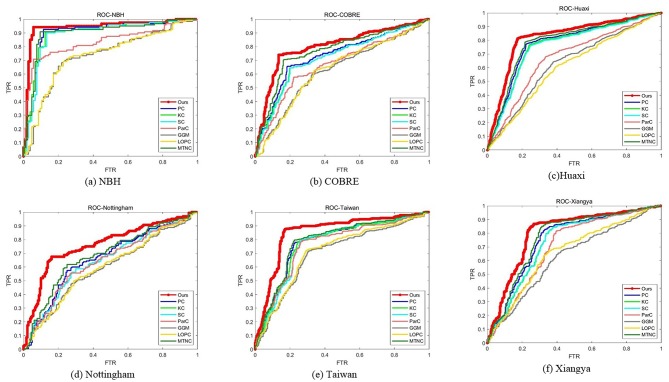
ROC curves for all methods on all datasets.

We report the comparison results of classification based on different functional brain networks on different datasets in Table 3. In particular, we also report the *p*-values and mark statistically significant differences (*p* < 0.05) with the asterisk (*). The receiver operating characteristic (ROC) curves are shown in Figure 3. It can be seen that our BFCN construction method has the best diagnostic results on six schizophrenia datasets among all brain network construction methods. Discussion of experimental results is given in section Discussion.

**Table 3:** Classification results (ACC/SEN/SPE/PPV/NPV/F1/BAC/AUC±STD%, and *p*-value) by *k*-fold cross-validation of several Functional fMRI networks algorithms on schizophrenia datasets.

		**ACC ± STD**	**SEN ± STD**	**SPE ± STD**	**PPV ± STD**	**NPV ± STD**	**F1 ± STD**	**BAC ± STD**	**AUC ± STD**	***p*-value**
NBH	PC	90.67 ± 1.66	93.59 ± 3.24	89.67 ± 2.80	91.12 ± 1.09	91.40 ± 5.34	91.33 ± 1.85	91.63 ± 1.16	88.41 ± 1.07	0.0330*
	KC	88.89 ± 1.41	91.18 ± 1.84	88.92 ± 3.68	90.21 ± 3.55	87.27 ± 2.49	89.59 ± 1.32	90.05 ± 1.31	84.84 ± 2.72	0.0208*
	SC	88.44 ± 1.66	91.18 ± 1.84	88.26 ± 2.58	89.88 ± 2.02	87.27 ± 2.49	89.28 ± 2.26	89.72 ± 1.38	84.21 ± 3.28	0.0068*
	ParC	81.78 ± 1.66	72.25 ± 4.48	94.93 ± 1.60	94.40 ± 2.04	76.06 ± 3.11	79.26 ± 3.29	83.59 ± 1.65	72.78 ± 3.29	0.0246*
	GGM	72.89 ± 1.66	68.75 ± 4.50	80.20 ± 4.75	82.29 ± 5.95	68.82 ± 3.15	72.16 ± 1.87	74.48 ± 0.92	71.19 ± 2.91	4.1573e-4*
	LOPC	72.89 ± 2.95	69.72 ± 4.63	78.73 ± 4.36	81.31 ± 4.97	74.08 ± 4.08	70.75 ± 3.70	74.23 ± 1.76	71.79 ± 2.99	3.7009e-4*
	MTNC	90.67 ± 1.66	91.99 ± 3.30	90.73 ± 2.98	92.69 ± 2.31	89.80 ± 5.22	91.38 ± 1.87	91.36 ± 1.12	87.70 ± 1.17	0.0462*
	Ours	**94.22** **±** **2.67**	**94.69** **±** **3.64**	**94.93** **±** **3.08**	**95.27** **±** **3.16**	**93.00** **±** **5.42**	**94.36** **±** **2.52**	**94.81** **±** **2.10**	**91.79** **±** **2.36**	–
COBRE	PC	74.50 ± 1.55	66.10 ± 2.22	82.66 ± 3.55	74.99 ± 2.84	75.43 ± 1.47	68.57 ± 0.92	74.38 ± 1.49	69.50 ± 1.47	0.0011*
	KC	73.17 ± 1.93	64.49 ± 1.20	81.11 ± 4.27	73.18 ± 3.23	74.24 ± 1.15	66.93 ± 1.06	72.80 ± 1.67	68.22 ± 1.02	6.4777e-4*
	SC	73.00 ± 1.63	64.49 ± 1.20	80.86 ± 3.52	72.98 ± 2.66	74.15 ± 1.16	66.83 ± 0.97	72.67 ± 1.37	68.13 ± 0.73	4.1727e-4*
	ParC	69.00 ± 1.93	59.16 ± 3.70	78.45 ± 1.93	69.31 ± 1.67	70.07 ± 1.67	62.22 ± 2.89	68.80 ± 1.92	62.87 ± 1.99	2.0603e-5*
	GGM	63.83 ± 2.56	60.82 ± 1.13	67.74 ± 4.16	60.94 ± 3.39	68.69 ± 1.23	58.85 ± 1.97	64.28 ± 1.99	59.02 ± 3.52	1.2521e-4*
	LOPC	64.33 ± 1.53	62.98 ± 4.59	65.85 ± 3.87	59.93 ± 2.40	68.96 ± 1.80	60.15 ± 2.62	64.41 ± 2.05	59.85 ± 3.01	2.0818e-4*
	MTNC	77.83 ± 1.45	70.80 ± 1.54	84.64 ± 2.75	78.16 ± 1.70	78.64 ± 1.29	72.70 ± 1.40	77.72 ± 1.28	72.71 ± 0.54	0.0098*
	Ours	**81.00** **±** **0.62**	**74.51** **±** **4.73**	**87.53** **±** **3.70**	**81.30** **±** **3.45**	**81.33** **±** **2.17**	**76.36** **±** **1.52**	**81.02** **±** **0.89**	**76.39** **±** **2.01**	–
Huaxi	PC	77.49 ± 0.88	77.44 ± 1.74	77.72 ± 2.37	79.03 ± 1.33	76.10 ± 1.02	77.88 ± 0.76	77.58 ± 0.96	77.10 ± 0.85	1.4912e-4*
	KC	76.28 ± 1.41	76.73 ± 1.46	75.98 ± 2.56	77.73 ± 1.62	74.97 ± 1.55	76.88 ± 1.09	76.36 ± 1.52	75.48 ± 1.35	7.7997e-4*
	SC	75.70 ± 1.73	76.14 ± 1.07	75.45 ± 3.01	77.18 ± 2.06	74.45 ± 1.76	76.29 ± 1.29	75.80 ± 1.85	74.89 ± 1.56	0.0011*
	ParC	67.20 ± 1.68	68.54 ± 2.39	66.84 ± 1.77	68.90 ± 1.70	66.47 ± 2.06	68.07 ± 1.78	67.69 ± 1.87	67.14 ± 1.58	1.8221e-5*
	GGM	63.10 ± 2.51	64.53 ± 3.02	61.33 ± 2.16	64.12 ± 1.98	61.81 ± 2.90	64.05 ± 2.52	62.93 ± 2.37	62.46 ± 3.21	6.8929e-5*
	LOPC	60.66 ± 1.36	60.52 ± 2.69	60.75 ± 1.08	62.28 ± 0.88	58.96 ± 1.48	61.05 ± 1.95	60.63 ± 1.03	59.90 ± 2.03	4.3858e-6*
	MTNC	78.52 ± 1.66	78.98 ± 2.26	78.23 ± 2.17	79.79 ± 1.43	77.43 ± 1.99	79.03 ± 1.65	78.61 ± 1.81	78.24 ± 1.20	0.0175*
	Ours	**81.94** **±** **0.74**	**81.56** **±** **1.44**	**82.71** **±** **1.98**	**83.50** **±** **1.53**	**80.42** **±** **1.31**	**82.24** **±** **0.65**	**82.14** **±** **1.66**	**81.25** **±** **0.98**	–
Nottingham	PC	66.31 ± 3.49	60.59 ± 7.84	72.84 ± 3.32	65.75 ± 5.86	68.21 ± 4.51	60.75 ± 6.30	66.74 ± 4.46	63.87 ± 3.18	9.5656e-5*
	KC	64.58 ± 2.89	59.36 ± 6.67	70.34 ± 3.43	63.43 ± 3.58	66.42 ± 3.54	59.26 ± 4.83	64.85 ± 3.78	61.89 ± 2.95	7.7695e-4*
	SC	64.58 ± 2.89	59.36 ± 6.67	70.34 ± 3.43	63.43 ± 3.58	66.42 ± 3.54	59.26 ± 4.83	64.85 ± 3.78	61.89 ± 2.95	7.7695e-4*
	ParC	64.63 ± 1.73	56.31 ± 2.90	73.79 ± 2.63	66.52 ± 4.59	65.32 ± 1.52	58.25 ± 3.11	65.05 ± 1.53	62.05 ± 3.58	3.6260e-4*
	GGM	60.19 ± 1.80	49.29 ± 3.64	69.19 ± 1.54	59.80 ± 3.00	60.40 ± 1.89	52.13 ± 2.43	59.27 ± 1.46	57.85 ± 4.08	0.0027*
	LOPC	60.56 ± 1.63	53.18 ± 5.26	67.09 ± 3.62	60.16 ± 3.52	61.25 ± 2.49	53.89 ± 1.67	60.13 ± 1.21	58.68 ± 3.45	0.0025*
	MTNC	68.77 ± 2.84	62.52 ± 7.58	75.83 ± 2.71	68.44 ± 5.27	70.41 ± 3.61	63.14 ± 6.04	69.17 ± 3.63	65.73 ± 2.61	5.1598e-4*
	Ours	**75.88** **±** **1.54**	**67.05** **±** **5.53**	**84.63** **±** **3.79**	**77.92** **±** **4.27**	**75.41** **±** **2.09**	**69.92** **±** **4.05**	**75.84** **±** **2.13**	**73.25** **±** **3.47**	–
Taiwan	PC	77.42 ± 2.30	77.83 ± 3.12	77.60 ± 3.49	79.32 ± 2.34	76.14 ± 3.41	77.83 ± 2.37	77.72 ± 2.53	77.07 ± 2.54	0.0027*
	KC	76.80 ± 1.48	78.43 ± 4.28	75.81 ± 3.03	77.92 ± 1.52	76.66 ± 3.34	77.39 ± 1.96	77.12 ± 1.81	76.11 ± 3.75	6.7739e-4*
	SC	76.80 ± 1.95	79.80 ± 4.03	74.36 ± 2.76	77.30 ± 1.74	77.32 ± 3.62	77.77 ± 2.13	77.08 ± 2.09	76.03 ± 3.21	0.0014*
	ParC	76.01 ± 1.50	79.80 ± 2.71	74.12 ± 2.56	76.94 ± 2.17	77.11 ± 1.69	77.14 ± 1.63	76.96 ± 1.23	75.18 ± 5.97	9.0876e-4*
	GGM	70.08 ± 1.76	71.84 ± 4.11	68.65 ± 3.72	70.76 ± 2.33	69.04 ± 2.00	70.65 ± 2.87	70.25 ± 2.33	69.78 ± 4.15	8.6965e-4*
	LOPC	71.15 ± 1.31	72.56 ± 3.37	69.89 ± 2.68	72.60 ± 1.90	69.79 ± 1.67	71.77 ± 1.82	71.22 ± 1.48	70.83 ± 2.82	0.0010*
	MTNC	78.50 ± 1.69	79.68 ± 2.73	78.02 ± 3.30	80.01 ± 1.93	77.74 ± 2.97	79.13 ± 1.62	78.85 ± 1.85	78.17 ± 3.02	0.0022*
	Ours	**85.17** **±** **0.78**	**88.06** **±** **1.74**	**82.73** **±** **0.17**	**84.91** **±** **0.94**	**85.58** **±** **2.36**	**85.99** **±** **0.69**	**85.39** **±** **0.83**	**83.71** **±** **4.12**	–
Xiangya	PC	75.93 ± 1.78	84.18 ± 1.43	65.40 ± 4.20	76.60 ± 3.06	75.36 ± 1.88	79.62 ± 1.90	74.79 ± 2.63	74.29 ± 2.52	8.7979e-5*
	KC	74.88 ± 1.93	84.30 ± 2.10	63.15 ± 2.79	75.67 ± 1.82	74.74 ± 2.69	79.07 ± 1.76	73.73 ± 2.15	72.33 ± 1.08	0.0021*
	SC	74.85 ± 1.55	83.89 ± 2.29	63.40 ± 2.07	75.64 ± 1.78	74.25 ± 2.61	78.94 ± 1.70	73.65 ± 1.87	72.22 ± 2.17	2.5002e-4*
	ParC	72.29 ± 1.78	83.28 ± 0.81	57.42 ± 4.19	72.83 ± 1.81	70.97 ± 1.37	77.21 ± 0.96	70.35 ± 1.88	70.11 ± 3.02	0.0057*
	GGM	63.09 ± 2.12	67.37 ± 2.54	56.91 ± 6.63	68.77 ± 2.76	54.93 ± 3.28	67.46 ± 1.88	62.14 ± 3.13	61.73 ± 4.71	2.6668e-4*
	LOPC	65.66 ± 1.87	66.54 ± 1.75	63.82 ± 4.30	72.03 ± 2.46	57.80 ± 2.75	68.59 ± 1.45	65.18 ± 2.38	65.56 ± 3.60	4.6110e-5*
	MTNC	78.06 ± 1.72	86.43 ± 2.72	67.62 ± 2.87	78.14 ± 2.26	78.29 ± 3.20	81.49 ± 2.05	77.03 ± 2.48	76.04 ± 3.14	0.0153*
	Ours	**80.83** **±** **1.02**	**85.98** **±** **3.17**	**74.47** **±** **3.35**	**81.41** **±** **2.08**	**79.70** **±** **2.73**	**83.19** **±** **1.63**	**80.23** **±** **1.28**	**79.20** **±** **2.89**	–

*The best results are in boldface*.

The asterisk (*) denotes the statistically significant differences (*p* < 0.05).

### Classification Performance of Different Dimension Reduction and Classification Methods

This experiment is designed to test the performance of our brain network under different dimensionality reduction and classification methods. In this experiment, the brain network is constructed using the proposed method. Some widely used feature extraction and classification methods in functional brain network analysis are compared.

#### Comparison Methods

We chose the following classifiers to conduct the experiment: nearest neighbor classifier (NN) without feature selection (All original features are adopted as the baseline in the experiments), linear discriminant analysis (LDA) (Zhang and Jia, 2007), Isometric feature mapping (ISOMAP) (Tenenbaum, 1998), neighborhood preserving embedding (NPE) (He et al., 2005), support machine vector (SVM) (Chang and Lin, 2011), and KDA (Cai et al., 2011).

#### Experimental Setup

Similar to the previous experiment, we adopt 10-fold cross-validation strategy and repeated this process 20 times. ACC, SEN, SPE, PPV, NPV, BAC, AUC, and their STD are employed to measure the performance in classification. *P*-value of the AUC is used to measure statistically significant differences between KDA and other feature extraction methods.

For fair comparison, for the generation of functional brain networks we adopt a grid search to select the parameter of the number of neighbors from {5, 10, 15, … , 45}, and the weighted coefficient of the hybrid brain network is selected from {0, 0.1, 0.2, … , 1}. We set threshold from {0, 0.05, 0.1, … , 0.4} for hybrid brain network on all datasets. For the feature selection algorithm, we use Gaussian kernel function in KDA. For the similarity-based graph methods, including ISOMAP and NPE, we build an edge between two subjects if and only if they belong to the same class. For SVM, we adopt linear kernel.

#### Results and Analysis

We report the comparison results of classification based on different feature selection and classification methods on different datasets in Table 4. In particular, we mark statistically significant differences (*p* < 0.05) with the asterisk (*). It can be seen that our method has the best ACC and AUC performance on six datasets compared to other feature selection and classification algorithms. Discussion of experimental results is given in section Discussion.

**Table 4:** Classification results (ACC/SEN/SPE/PPV/NPV/F1/BAC/AUC±STD%, and *p*-value) by *k*-fold cross-validation of several feature selection algorithms on schizophrenia datasets.

		**ACC ± STD**	**SEN ± STD**	**SPE ± STD**	**PPV ± STD**	**NPV ± STD**	**F1 ± STD**	**BAC ± STD**	**AUC ± STD**	***p*-value**
NBH	NN	83.11 ± 3.01	73.61 ± 7.87	**96.53** **±** **2.96**	**96.27** **±** **3.90**	75.29 ± 4.01	81.66 ± 3.90	85.07 ± 2.56	83.02 ± 2.14	0.0174*
	LDA	91.56 ± 1.66	93.23 ± 2.73	90.67 ± 3.87	93.35 ± 2.79	91.13 ± 4.14	92.63 ± 1.30	91.95 ± 1.81	88.41 ± 2.76	0.0264*
	ISOMAP	86.67 ± 3.14	84.20 ± 8.21	89.73 ± 6.23	90.63 ± 4.08	82.95 ± 6.37	86.14 ± 3.83	86.96 ± 3.77	83.29 ± 2.61	0.0029*
	NPE	91.11 ± 1.41	85.20 ± 3.75	96.40 ± 3.67	96.86 ± 2.58	88.80 ± 2.04	89.28 ± 2.28	90.80 ± 1.02	87.38 ± 1.34	0.0269*
	SVM	92.00 ± 2.27	94.35 ± 3.54	91.16 ± 3.54	92.26 ± 2.07	92.40 ± 4.87	92.46 ± 2.46	92.76 ± 2.07	88.97 ± 2.69	0.0152*
	Ours	**94.22** **±** **2.67**	**94.69** **±** **3.64**	94.93 ± 3.08	95.27 ± 3.16	**93.00** **±** **5.42**	**94.36** **±** **2.52**	**94.81** **±** **2.10**	**91.79** **±** **2.36**	–
COBRE	NN	66.67 ± 2.17	38.64 ± 1.89	**88.55** **±** **2.52**	70.25 ± 6.41	64.88 ± 1.36	48.82 ± 3.10	63.59 ± 2.04	59.84 ± 5.33	0.0013*
	LDA	77.00 ± 1.94	70.72 ± 1.20	83.30 ± 3.22	77.05 ± 1.75	78.41 ± 1.37	72.01 ± 1.25	77.01 ± 1.45	71.99 ± 0.64	0.0109*
	ISOMAP	72.50 ± 2.17	57.20 ± 5.09	86.47 ± 2.23	77.27 ± 3.15	72.10 ± 3.02	63.24 ± 3.07	71.84 ± 2.66	66.26 ± 3.68	4.3278e-4*
	NPE	73.83 ± 2.33	79.86 ± 6.41	68.20 ± 6.70	67.99 ± 3.09	82.98 ± 5.92	71.78 ± 2.87	74.03 ± 2.32	71.57 ± 2.64	0.0404*
	SVM	77.17 ± 1.25	68.67 ± 2.41	84.62 ± 2.07	77.66 ± 1.35	77.77 ± 1.10	71.41 ± 1.41	76.65 ± 1.01	72.66 ± 1.11	0.0035*
	Ours	**81.00** **±** **0.62**	**74.51** **±** **4.73**	87.53 ± 3.70	**81.30** **±** **3.45**	**81.33** **±** **2.17**	**76.36** **±** **1.52**	**81.02** **±** **0.89**	**76.39** **±** **2.01**	–
Huaxi	NN	63.28 ± 1.06	58.91 ± 3.63	68.59 ± 3.42	66.43 ± 1.36	61.06 ± 1.03	61.90 ± 2.13	63.75 ± 0.94	62.77 ± 1.47	1.5948e-6*
	LDA	77.75 ± 1.33	78.10 ± 1.70	77.64 ± 2.27	79.12 ± 1.39	76.58 ± 1.36	78.25 ± 1.24	77.87 ± 1.46	77.36 ± 1.22	7.0516e-4*
	ISOMAP	70.11 ± 1.92	62.20 ± 7.10	78.75 ± 5.60	76.20 ± 3.05	66.66 ± 3.18	67.59 ± 3.44	70.48 ± 1.67	69.76 ± 1.31	3.7721e-4*
	NPE	73.98 ± 0.40	67.52 ± 3.24	79.02 ± 4.32	79.15 ± 2.18	70.66 ± 1.88	72.00 ± 1.17	73.27 ± 0.89	74.20 ± 1.69	0.0010*
	SVM	80.58 ± 0.74	81.33 ± 0.70	80.08 ± 1.49	81.36 ± 0.86	79.93 ± 0.83	81.05 ± 0.64	80.71 ± 0.91	79.93 ± 1.52	0.0339*
	Ours	**81.94** **±** **0.74**	**81.56** **±** **1.44**	**82.71** **±** **1.98**	**83.50** **±** **1.53**	**80.42** **±** **1.31**	**82.24** **±** **0.65**	**82.14** **±** **1.66**	**81.25** **±** **0.98**	–
Nottingham	NN	68.58 ± 2.53	56.62 ± 12.24	80.06 ± 7.84	71.97 ± 3.40	67.60 ± 4.37	60.77 ± 7.15	68.34 ± 2.60	66.79 ± 4.48	0.0094*
	LDA	68.71 ± 2.87	62.92 ± 7.48	74.83 ± 4.50	68.57 ± 5.14	70.21 ± 3.72	63.33 ± 5.97	68.87 ± 3.96	65.52 ± 2.31	9.7785e-4*
	ISOMAP	69.75 ± 2.75	57.31 ± 9.18	81.81 ± 9.65	76.94 ± 4.91	67.69 ± 2.59	62.75 ± 5.78	69.56 ± 3.11	67.53 ± 5.70	0.0369*
	NPE	72.83 ± 1.37	**75.20** **±** **4.52**	66.84 ± 3.22	71.09 ± 2.58	**77.51** **±** **2.40**	71.12 ± 2.62	71.02 ± 3.06	69.74 ± 1.34	0.0340*
	SVM	75.73 ± 2.85	70.28 ± 3.18	82.11 ± 5.55	**78.89** **±** **5.37**	75.76 ± 1.95	**72.37** **±** **2.82**	**76.20** **±** **2.44**	71.16 ± 3.89	0.0256*
	Ours	**75.88** **±** **1.54**	67.05 ± 5.53	**84.63** **±** **3.79**	77.92 ± 4.27	75.41 ± 2.09	69.92 ± 4.05	75.84 ± 2.13	**73.25** **±** **3.47**	–
Taiwan	NN	73.75 ± 0.57	77.25 ± 3.99	71.26 ± 3.26	74.51 ± 1.27	73.94 ± 2.85	74.98 ± 1.39	74.25 ± 1.17	72.47 ± 3.97	1.8216e-4*
	LDA	79.85 ± 2.38	83.47 ± 4.87	77.09 ± 2.96	80.28 ± 1.72	81.29 ± 3.48	80.90 ± 2.46	80.28 ± 3.00	78.97 ± 3.42	0.0129*
	ISOMAP	75.28 ± 1.05	72.68 ± 1.71	79.86 ± 2.96	79.46 ± 2.09	72.37 ± 1.47	74.89 ± 0.89	76.27 ± 1.99	75.29 ± 3.74	6.6160e-4*
	NPE	79.57 ± 0.86	73.90 ± 5.45	**83.19** **±** **5.32**	**86.47** **±** **4.03**	74.78 ± 0.91	78.26 ± 2.00	78.55 ± 0.79	77.27 ± 1.10	0.0396*
	SVM	79.84 ± 1.63	82.99 ± 3.25	77.78 ± 3.16	80.57 ± 1.77	80.57 ± 2.64	80.89 ± 1.51	80.38 ± 2.24	78.91 ± 3.45	0.0012*
	Ours	**85.17** **±** **0.78**	**88.06** **±** **1.74**	82.73 ± 0.17	84.91 ± 0.94	**85.58** **±** **2.36**	**85.99** **±** **0.69**	**85.39** **±** **0.83**	**83.71** **±** **4.12**	–
Xiangya	NN	65.79 ± 1.37	76.99 ± 8.68	51.05 ± 7.96	68.65 ± 1.50	63.23 ± 5.85	71.47 ± 3.45	64.02 ± 1.38	62.80 ± 3.34	4.4581e-6*
	LDA	77.64 ± 1.27	84.90 ± 2.51	68.07 ± 2.96	78.33 ± 2.09	76.72 ± 1.21	80.92 ± 1.74	76.49 ± 1.44	75.71 ± 2.46	0.0035*
	ISOMAP	66.52 ± 1.23	74.39 ± 2.55	56.65 ± 1.59	70.04 ± 1.74	61.31 ± 2.12	71.46 ± 1.79	65.52 ± 1.72	65.33 ± 2.37	6.5089e-5*
	NPE	72.05 ± 1.76	63.22 ± 4.17	80.25 ± 5.04	83.16 ± 3.62	62.32 ± 2.19	70.77 ± 2.05	71.74 ± 1.28	73.02 ± 1.99	0.0367*
	SVM	78.23 ± 1.63	**86.16** **±** **1.95**	67.63 ± 2.48	77.97 ± 2.18	77.92 ± 2.07	81.48 ± 1.99	76.89 ± 2.13	75.78 ± 3.05	0.0031*
	Ours	**80.83** **±** **1.02**	85.98 ± 3.17	**74.47** **±** **3.35**	**81.41** **±** **2.08**	**79.70** **±** **2.73**	**83.19** **±** **1.63**	**80.23** **±** **1.28**	**79.20** **±** **2.89**	–

*The best results are in boldface*.

The asterisk (*) denotes the statistically significant differences (*p* < 0.05).

## Discussion

In the first experiment, we compared the performance of our functional networks and other functional networks in ACC, SEN, SPE, PPV, NPV, BAC, and AUC on six schizophrenia datasets and reported the *p*-value of the AUC between proposed method and comparison methods. Experimental results have proved that our functional network is effective and superior to other functional networks in all the measures and the results are significantly different. In addition, we draw ROC curves of our method and all comparison methods on each dataset. It can be seen that the ROC curves of the comparison methods are almost at the bottom right of the ROC curve of our method, and the results of the area under the curve of the comparison methods are also smaller than our method. The reason why our method can show the above performance may be that our network not only considers the relationship between the two brain regions, but also preserves the second-order information among brain regions. First-order information has a certain robustness, and second-order information is more sensitive to signals. In the comparison method, PC, KC, SC, and MTNC only consider the first-order information, and ParC, GGM, and LOPC only consider the second-order information. Our hybrid networks combining the two types of information may achieve better results.

In the second experiment, we compared the performance of our network with several classification algorithms. The evaluation indicators are ACC, SEN, SPE, PPV, NPV, BAC, and AUC. In addition, *p*-values of the AUC between proposed method and comparison methods are also reported. The experiment proves that our hybrid brain network is robust and performs well under each classifier. In addition, the experimental results show that our framework performs well, and the ACC and AUC indicators have achieved the best results on the six schizophrenia datasets. This may be due to the complexity of the brain network, which is not simply linearly separable, and our framework chose the KDA classifier, which can select the most significant connectivities and improve the classification performance.

In addition to classification performance, there are many other indicators that evaluate the functional brain networks. The topology metric of the network is a widely used one. We compared the performance of our network and PC based method with some topological properties, and calculated the corresponding *p*-value, including connectivity strength, average degree, density, clustering coefficient, characteristic path length, global efficiency, local efficiency, closeness centrality, edge betweenness centrality, node betweenness centrality, radiality, assortativity, structural consistency, and fitted exponent of power-law degree distribution. The brain network that calculates the topological properties consists of the brain networks that obtained the best classification performance in Experiment 3.1. The experimental results are shown in Table 5. The asterisk (*) denotes the statistically significant differences (*p* < 0.05).

**Table 5:** The topological properties of our proposed method and PC based method.

	**Topology metrics**	**Healthy controls (mean ± std)**	**Schizophrenia patients (mean ±, std)**	***p*-value**
Proposed method	Connectivity strength	32.3520 ± 1.6443	31.5238 ± 1.5095	0.0430*
	Average degree	87.0486 ± 0.9023	86.5014 ± 1.2570	0.0323*
	Density	0.9781 ± 0.0101	0.9719 ± 0.0141	0.0323*
	Clustering coefficient	0.9802 ± 0.0083	0.9755 ± 0.0102	0.0295*
	Characteristic path length	1.0219 ± 0.0101	1.0281 ± 0.0141	0.0323*
	global efficiency	0.9890 ± 0.0051	0.9860 ± 0.0071	0.0323*
	Local efficiency	0.9901 ± 0.0042	0.9877 ± 0.0051	0.0295*
	Closeness centrality	88.0243 ± 0.4511	87.7507 ± 0.6285	0.0323*
	Edge betweenness centrality	1.0451 ± 0.0213	1.0582 ± 0.0309	0.0338*
	Node betweenness centrality	1.9514 ± 0.9023	2.4986 ± 1.2570	0.0323*
	Radiality	1.9781 ± 0.0101	1.9719 ± 0.0141	0.0323*
	Assortativity	−0.1272 ± 0.0447	−0.1515 ± 0.0385	0.0124*
	Structural consistency	0.9580 ± 0.0053	0.9613 ± 0.0050	0.0058*
	Fitted exponent of power-law degree distribution	8.4238 ± 0.0879	8.4868 ± 0.1182	0.0203*
Pearson based method	Connectivity strength	32.4749 ± 1.8836	31.5293 ± 1.5712	0.0893
	Average degree	88.7153 ± 0.0663	88.6889 ± 0.0570	0.5642
	Density	0.9968 ± 7.4499*e*−4	0.9969 ± 6.4031*e*−4	0.5642
	Clustering coefficient	0.9968 ± 7.5481*e*−4	0.9969 ± 6.4794*e*−4	0.5630
	Characteristic path length	1.0032 ± 7.4499*e*−4	1.0031 ± 6.4031*e*−4	0.5642
	Global efficiency	0.9984 ± 3.7249*e*−4	0.9984 ± 3.2016*e*−4	0.5642
	Local efficiency	0.9984 ± 3.7740*e*−4	0.9984 ± 3.2397*e*−4	0.5630
	Closeness centrality	88.8576 ± 0.0332	88.8620 ± 0.0285	0.5642
	Edge betweenness centrality	1.0064 ± 0.0015	1.0062 ± 0.0013	0.5636
	Node betweenness centrality	0.2847 ± 0.0663	0.2759 ± 0.0570	0.5642
	Radiality	1.9968 ± 7.4499*e*−4	1.9969 ± 6.4031*e*−4	0.5642
	Assortativity	−0.0195 ± 7.6192*e*−4	−0.0196 ± 6.5486*e*−4	0.5642
	Structural consistency	0.9700 ± 0.0068	0.9709 ± 0.0058	0.5621
	Fitted exponent of power-law degree distribution	8.3573 ± 3.5527*e*−15	8.3573 ± 1.7764*e*−15	NaN

The asterisk (*) denotes the statistically significant differences (p < 0.05).

The experimental results show that, compared to the PC based method, the brain network based on our functional brain network has better separability in these topological properties between the patient group and the healthy group. Specifically, we found that based on our proposed network, the connectivity strength of the health group is 32.3520 and that of the patient group is 31.5238. The functional network connectivity strength of the patient group is reduced by 0.8282, and there are similar findings in the literature (Shen et al., 2010). We also found that the clustering coefficient of the patient group was lower than that of the healthy group, which was also supported by the work of (Bachiller et al., 2014). Also, the global efficiency of patients with schizophrenia has decreased, indicating that the topology of the brain structure network in schizophrenia is less efficient. Griffa et al. (2014) has also mentioned this point.

**Figure 4:**
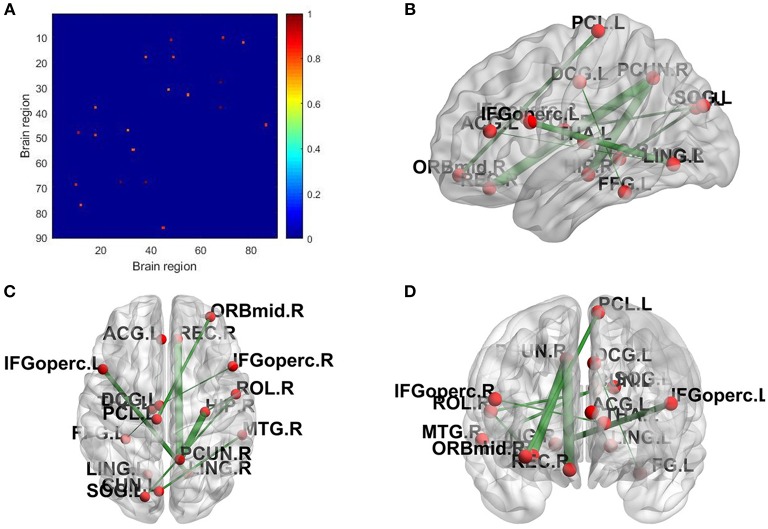
SAC between schizophrenia and healthy controls (Top 10). Brain network connectivities with the top 10 discrimination ability. In **(A)**, the position in the *i*-th row and *j*-th column indicates connectivity between the *i*-th brain region and the *j*-th brain region, and the weight can be judged by the corresponding color. **(B–D)** show the top 10 alteration connectivities for identifying schizophrenia.

The topological properties of the network show the differences in the overall brain network between the patient group and the health group. We also show the local difference with 30 connectivities that have the greatest differences in the patient group and the health group by calculating the significant alteration of connectivity (SAC). The results are shown in Table 6. To visualize these connectivities, we show the top 10 SAC drawn in Figure 4.

**Table 6:** SAC between schizophrenia and healthy controls (Top 30).

**No**.	**Brain region A**	**Brain region B**	**Weight score**
SAC 1	R. Precuneus	R. Rectus gyrus	1.0000
SAC 2	R. Precuneus	R. Hippocampus	0.9752
SAC 3	R. Lingual gyrus	L. Inferior frontal gyrus (opercular)	0.8986
SAC 4	L. Paracentral lobule	R. Orbitofrontal cortex (middle)	0.8532
SAC 5	R. Middle temporal gyrus	L. Cuneus	0.8304
SAC 6	L. Superior occipical gyrus	R. Rolandic operculum	0.8214
SAC 7	R. Hippocampus	R. Rolandic operculum	0.8121
SAC 8	L. Thalamus	R. Inferior frontal gyrus (opercular)	0.7993
SAC 9	L. Fusiform gyrus	L. Middle cingulate gyrus	0.7869
SAC 10	L. Lingual gyrus	L. Anterior cingulate gyrus	0.7768
SAC 11	R. Temporal pole (middle)	R. Middle temporal gyrus	0.7732
SAC 12	L. Paracentral lobule	L. Middle cingulate gyrus	0.7710
SAC 13	R. Middle cingulate gyrus	R. Olfactory	0.7645
SAC 14	R. Middle cingulate gyrus	L. Suplementary motor area	0.7436
SAC 15	R. Superior occipical gyrus	L. Suplementary motor area	0.7422
SAC 16	L. Heschl gyrus	R. Orbitofrontal cortex (superior)	0.7419
SAC 17	L. Angular gyrus	L. Superior frontal gyrus (dorsal)	0.7415
SAC 18	L. Rectus gyrus	L. Superior frontal gyrus (dorsal)	0.7344
SAC 19	L. Precuneus	L. Superior parietal gyrus	0.7245
SAC 20	L. Thalamus	R. Amygdala	0.7227
SAC 21	R. Inferior temporal	L. Rolandic operculum	0.7054
SAC 22	L. Superior frontal gyrus (media)	R. Orbitofrontal cortex (superior)	0.6965
SAC 23	L. Inferior temporal	L. Rectus gyrus	0.6941
SAC 24	L. Heschl gyrus	R. Insula	0.6937
SAC 25	L. Precuneus	L. Precentral gyrus	0.6892
SAC 26	R. Pallidum	R. Amygdala	0.6884
SAC 27	R. Thalamus	R. Calcarine cortex	0.6855
SAC 28	R. Angular gyrus	L. Fusiform gyrus	0.6788
SAC 29	L. Postcentral gyrus	R. Anterior cingulate gyrus	0.6704
SAC 30	R. Inferior temporal	R. Cuneus	0.6695

**Figure 5:**
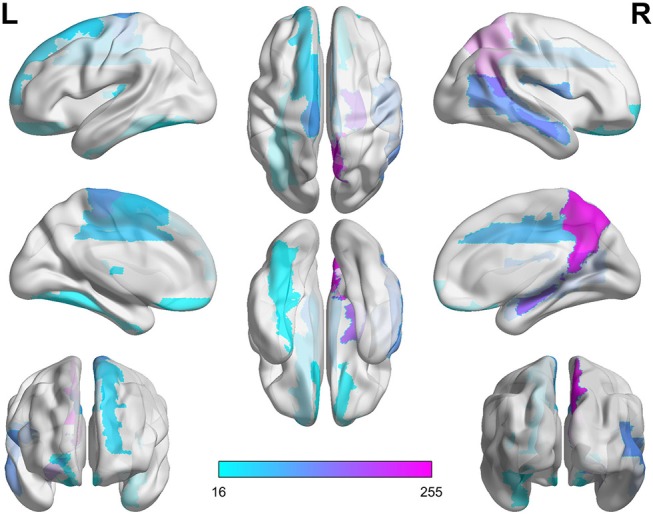
Significant alteration of brain regions between schizophrenia and healthy controls.

As shown in Table 6 and Figure 4, the connectivity between Precuneus and other brain regions altered visibly, and (Faget-Agius et al., 2012) supports our finding. The connectivity between Hippocampus and other brain regions and the connectivity between Inferior frontal gyrus also have visible alteration, and there exist similar findings in some literature (Altshuler, 1990; Zhou et al., 2008; Kubicki et al., 2011). From Figure 4 and Table 6, it also can be seen that some brain regions are recurring, indicating that these brain regions play a key role in the analysis of differences between the patient group and the healthy group. Therefore, for each recurring brain region, we accumulate the weights of the SACs, where the brain region is located, as the weight of the brain region. All weights are normalized and sorted, and we list the top 15 important brain regions in Table 7 and visualize them in Figure 5.

**Table 7:** Significant alteration of brain regions between schizophrenia and healthy controls.

**No**.	**Brain region**	**Weight score**
ROI 1	R. Precuneus	1.0000
ROI 2	R. Hippocampus	0.6869
ROI 3	R. Rolandic operculum	0.4307
ROI 4	L. Paracentral lobule	0.4152
ROI 5	R. Middle temporal gyrus	0.3809
ROI 6	L. Middle cingulate gyrus	0.3048
ROI 7	L. Thalamus	0.2450
ROI 8	R. Middle cingulate gyrus	0.2218
ROI 9	L. Suplementary motor area	0.1847
ROI 10	L. Superior frontal gyrus (dorsal)	0.1682
ROI 11	L. Fusiform gyrus	0.1512
ROI 12	R. Orbitofrontal cortex (superior)	0.1057
ROI 13	L. Heschl gyrus	0.1011
ROI 14	L. Rectus gyrus	0.0892
ROI 15	L. Precuneus	0.0646

As shown in Table 7 and Figure 5, some brain regions such as Precuneus, Hippocampus and Rolandic operculum are selected. This suggests that these brain regions play an important role in the task of identifying patients with schizophrenia and normal controls, and that these brain regions may be important biomarkers for schizophrenia. Some literatures (Bettus et al., 2009; Salvador et al., 2010; Faget-Agius et al., 2012; Qiu et al., 2018) also prove our findings.

**Figure 6:**
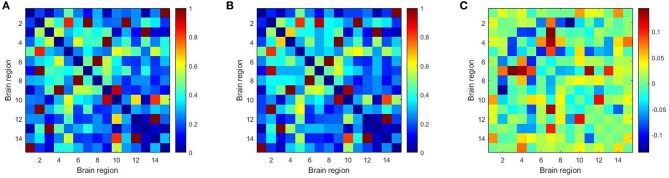
The average patient group subnet **(A)**, average health group subnet **(B)**, the difference between average patient group subnets and average health group subnets **(C)**.

Furthermore, we constructed the connectivity subnets with these 15 brain regions in Table 7. The average patient group subnet, average health group subnet, the difference between average patient group subnet and average health group subnet are shown in Figure 6. It can be seen that although the strength of the patient group connectivity on the overall network is lower than that of the healthy group, the average connectivity strength of the patient group in the subnet is slightly higher than that of the healthy group. This change further demonstrates the importance of the brain regions we have discovered in identifying tasks in patients with schizophrenia, that is, these brain regions may play an important role in schizophrenia classification.

## Conclusion and Future Work

In summary, we propose a hybrid functional brain network with first-order and second-order information for identifying schizophrenia. Specifically, we construct a second-order brain network through triplet correlation, and fuse it with conventional first-order brain network. The second-order brain network is more sensitive to the difference in brain networks between patients and healthy controls, and the first-order brain network is more robust to noise. The proposed method not only captures higher-order information among brain regions, but also reveals the ordinal information of connectivity strength between specific brain regions. Experiments on six schizophrenia datasets show that our method is superior to the existing BFCN construction method. In addition, we analyzed the differences in topological properties between schizophrenia patients and normal controls with the proposed brain network.

This study uses the grid search method in the process of constructing the hybrid functional brain network with first- and second-order information. We will investigate more efficient parameter selection methods in future work. In addition, this study is conducted separately on a dataset from a single site. In our future work, we will focus on how to construct and analyze the high-order brain network on the cross-site dataset, which is helpful to improve the robustness of the model.

## Author Contributions

QZ conceived the experiment and revised the manuscript. HL was responsible for the experiment and writing the first draft of the manuscript. JH and DG were responsible for preprocessing the fMRI data. XX gave clinical guidance for biomarkers. DZ contributed to data analysis. All authors read and endorsed the final draft.

### Conflict of Interest Statement

The authors declare that the research was conducted in the absence of any commercial or financial relationships that could be construed as a potential conflict of interest. The reviewer YW declared a past co-authorship with one of the authors DZ to the handling editor.
